# The effect of community-driven larval source management and house improvement on malaria transmission when added to the standard malaria control strategies in Malawi: a cluster-randomized controlled trial

**DOI:** 10.1186/s12936-021-03769-0

**Published:** 2021-05-22

**Authors:** Robert S. McCann, Alinune N. Kabaghe, Paula Moraga, Steven Gowelo, Monicah M. Mburu, Tinashe Tizifa, Michael G. Chipeta, William Nkhono, Aurelio Di Pasquale, Nicolas Maire, Lucinda Manda-Taylor, Themba Mzilahowa, Henk van den Berg, Peter J. Diggle, Dianne J. Terlouw, Willem Takken, Michèle van Vugt, Kamija S. Phiri

**Affiliations:** 1 Laboratory of Entomology, Wageningen University & Research, Wageningen, The Netherlands; 2 School of Public Health and Family Medicine, College of Medicine, University of Malawi, Blantyre, Malawi; 3 Center for Vaccine Development and Global Health, University of Maryland School of Medicine, Baltimore, USA; 4 Center for Tropical Medicine & Travel Medicine, Department of Infectious Diseases, Division of Internal Medicine, University of Amsterdam, Amsterdam, The Netherlands; 5 CHICAS, Lancaster Medical School, Lancaster University, Lancaster, UK; 6 Computer, Electrical and Mathematical Sciences and Engineering Division, King Abdullah University of Science and Technology, Thuwal, Saudi Arabia; 7 Big Data Institute, University of Oxford, Oxford, UK; 8 Malawi-Liverpool Wellcome Trust Clinical Research Program, Blantyre, Malawi; 9 Department of Epidemiology and Public Health, Swiss Tropical and Public Health Institute, Basel, Switzerland; 10 University of Basel, Basel, Switzerland; 11 MAC Communicable Diseases Action Centre, College of Medicine, University of Malawi, Blantyre, Malawi; 12 Clinical Sciences Department, Liverpool School of Tropical Medicine, Liverpool, UK

**Keywords:** Malaria, Larval source management, House improvement, Community engagement, Cluster randomised trial

## Abstract

**Background:**

Current standard interventions are not universally sufficient for malaria elimination. The effects of community-based house improvement (HI) and larval source management (LSM) as supplementary interventions to the Malawi National Malaria Control Programme (NMCP) interventions were assessed in the context of an intensive community engagement programme.

**Methods:**

The study was a two-by-two factorial, cluster-randomized controlled trial in Malawi. Village clusters were randomly assigned to four arms: a control arm; HI; LSM; and HI + LSM. Malawi NMCP interventions and community engagement were used in all arms. Household-level, cross-sectional surveys were conducted on a rolling, 2-monthly basis to measure parasitological and entomological outcomes over 3 years, beginning with one baseline year. The primary outcome was the entomological inoculation rate (EIR). Secondary outcomes included mosquito density, *Plasmodium falciparum* prevalence, and haemoglobin levels. All outcomes were assessed based on intention to treat, and comparisons between trial arms were conducted at both cluster and household level.

**Results:**

Eighteen clusters derived from 53 villages with 4558 households and 20,013 people were randomly assigned to the four trial arms. The mean nightly EIR fell from 0.010 infectious bites per person (95% CI 0.006–0.015) in the baseline year to 0.001 (0.000, 0.003) in the last year of the trial. Over the full trial period, the EIR did not differ between the four trial arms (p = 0.33). Similar results were observed for the other outcomes: mosquito density and *P. falciparum* prevalence decreased over 3 years of sampling, while haemoglobin levels increased; and there were minimal differences between the trial arms during the trial period.

**Conclusions:**

In the context of high insecticide-treated bed net use, neither community-based HI, LSM, nor HI + LSM contributed to further reductions in malaria transmission or prevalence beyond the reductions observed over two years across all four trial arms. This was the first trial, as far as the authors are aware, to test the potential complementary impact of LSM and/or HI beyond levels achieved by standard interventions. The unexpectedly low EIR values following intervention implementation indicated a promising reduction in malaria transmission for the area, but also limited the usefulness of this outcome for measuring differences in malaria transmission among the trial arms.

*Trial registration* PACTR, PACTR201604001501493, Registered 3 March 2016, https://pactr.samrc.ac.za/.

**Supplementary Information:**

The online version contains supplementary material available at 10.1186/s12936-021-03769-0.

## Background

The global burden of malaria was dramatically reduced from 2000 to 2015 [1]. In Africa, where 92% of malaria cases occur, this reduction was largely attributed to vector control with insecticide-treated bed nets (ITNs) and indoor residual spraying (IRS), and treatment with artemisinin-based combination therapy [2]. Yet progress in malaria control stalled in the period 2015–2017, with an estimated 219 million malaria cases occurring worldwide annually [3]. There are multiple, overlapping challenges limiting the ability of the current malaria interventions to continue reducing malaria burden at the rate observed from 2000 to 2015, including insecticide resistance [4], drug resistance [5], barriers to achieving target access, use, acceptability and sustainability [6], and residual malaria transmission [7].

Due to these well documented challenges, the need for additional malaria interventions is widely recognized [8]. In most cases, it is envisaged that additional interventions need to be deployed alongside existing core interventions, potentially leading to additive or synergistic effects beyond that of the core interventions on their own. Two vector control interventions that could provide additional protection from malaria when integrated into standard national control programmes are house improvement (HI) and larval source management (LSM) [9].

HI includes any structural modifications that reduce or eliminate mosquito house entry, such as plastering or screening walls, ceilings, eaves, windows or doors. The modifications may include a chemical component that repels or kills mosquitoes, such as eave baffles or eave tubes [10, 11], but they can also be entirely mechanical barriers. LSM includes any changes to water bodies that are potential larval habitats of mosquitoes to prevent the completion of development of the immature stages [12]. In recent decades, larval source management has most commonly consisted of either applying larvicides to potential larval habitats, or permanently removing the water bodies through draining or filling.

HI and LSM have both been associated with reducing malaria transmission in a wide variety of settings historically [12, 13]. More recently, improved housing resulting from more favourable social and economic conditions (in this case defined as houses built using finished wall, roof, and floor materials [14]) was associated with lower malaria parasite infection rates across the full range of malaria endemicity in Africa [14]. Two controlled trials of house improvement reporting epidemiological outcomes have been conducted in Africa [15, 16]. In both trials, the interventions were randomized at the household level, and house improvement reduced the number of *Anopheles* mosquitoes indoors. HI reduced the risk of anaemia in children in The Gambia [15] and the incidence of malaria cases in children in Ethiopia [16]. Results of recent controlled trials of LSM on epidemiological outcomes in Africa have been mixed, suggesting that the impact is dependent on both the larval ecology of the local vector mosquitoes and the implementation strategy [17].

Vector control interventions have a higher potential for success when community engagement and participation are explicitly incorporated into the control programme [18]. Community involvement can increase local programme ownership, encourage greater awareness of health promotion, and increase uptake and sustainability of interventions [19, 20]. HI and LSM require a considerable labour input, and both interventions are essentially interwoven into a community via their living space and surrounding environment. Therefore, integrating a strong community engagement strategy into the implementation of these interventions could strengthen their effectiveness, scalability, and sustainability.

This study aimed to inform malaria control policy in Malawi by evaluating the effect of community-based HI and LSM on malaria parasite prevalence and transmission intensity as measured by the entomological inoculation rate (EIR). The trial interventions, HI and LSM, were implemented as supplementary interventions in addition to the Malawi National Malaria Control Programme (NMCP) interventions, and the entire study site was part of an intensive community education and engagement programme aimed at increasing community participation in malaria control. The trial used a factorial design to evaluate the effects of HI and LSM alone or in combination with each other, and the outcomes were evaluated over a 24-month period.

## Methods

### Study design and participants

**Fig. 1:**
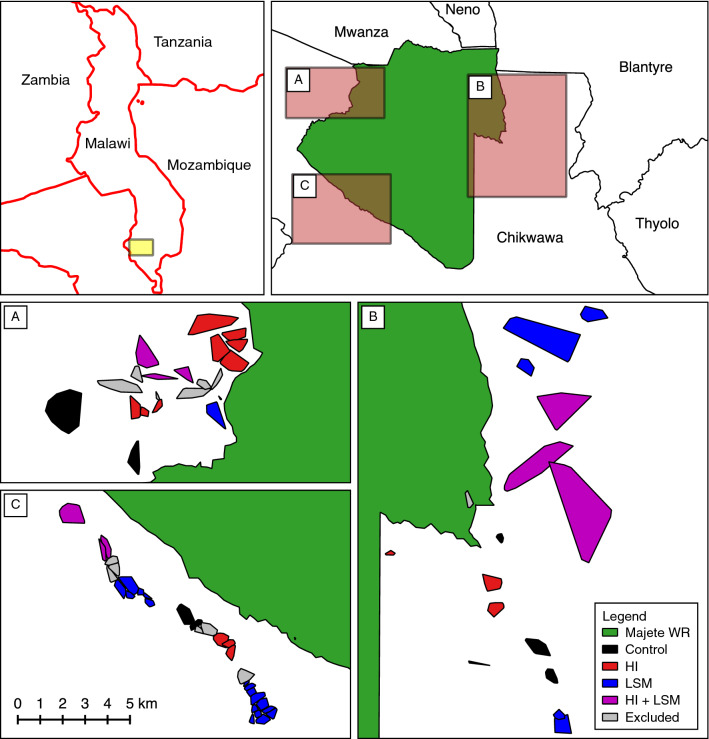
Maps of the study site. Top left panel shows the location of the site as a yellow rectangle in southern Malawi; country borders are shown in red. Top right panel shows locations of the three focal areas, labelled **A**, **B**, and **C**, around the perimeter of the Majete Wildlife Reserve (Majete WR); district borders are shown in black. **A**, **B**, and **C** show the locations of the 65 villages in the Majete Malaria Project catchment area, with the colour of each village denoting the trial arm allocation as indicated in the legend; scale bar applies to **A**, **B**, and **C**. *HI* house improvement, *LSM* larval source management

The study was a two-by-two factorial, cluster-randomized controlled trial in communities around the Majete Wildlife Reserve in Chikwawa District, southern Malawi (Fig. 1), an area that historically has high malaria transmission [21]. Malaria control in the district is based on the NMCP strategy and implemented through the Chikwawa District Health Office. From 2015 to 2018 the malaria control strategy consisted of provision of ITNs to pregnant women and children under 5 years old, mass distribution campaigns of ITNs, intermittent preventative therapy for pregnant women, and malaria case diagnosis and treatment with artemisinin-based combination therapy. The last mass distribution of ITNs in the district during the study period occurred in April 2016 and included PermaNet^®^ 2.0 (Vestergaard Frandsen, Lausanne, Switzerland), Olyset^®^ Net (Sumitomo Chemical Company, Tokyo, Japan), and Royal Sentry^®^ (Disease Control Technologies, USA). The NMCP implemented IRS in Chikwawa District in 2010 and 2012 with alphacypermethrin, but indoor residual spraying was not done in the district during the study period.

The trial villages were all located within the catchment area of the Majete Malaria Project, a collaboration of academic institutions, non-governmental organizations, and the government of Malawi. Community engagement and participation were a central focus of the project’s strategy to reduce malaria transmission and burden. The Hunger Project, a non-governmental organization specializing in community-based rural development, guided the principles followed to implement behaviour change communication (including fortnightly malaria workshops) and malaria vector control interventions through a community-based approach [22]. The trial was conducted in three regions, which were referred to as focal areas A, B and C (Fig. 1), covering a total population of about 25,000 people in 65 villages. The trial interventions were implemented at the village level, meaning that the entire population of each village was the target population.

There was considerable involvement of the Ministry of Health, the NMCP, and the District Health Office in the activities for the trial. Sensitization meetings were initially held at the district, traditional authority, group-village, and village administrative levels to inform community leaders and stakeholders about the intended trial, enabling the researchers to formally begin the process of community engagement for active community involvement and participation in the trial. Community engagement was an on-going process involving discussions between research staff, community leaders, and community members at the focal area and village levels. Community permission to conduct the trial was obtained from each village headman verbally, which was considered a culturally appropriate manner for the permission [23]. Community permission covered all aspects of the trial, including the implementation of the proposed trial interventions and activities for assessment of trial outcomes.

### Randomization

The trial interventions were implemented at the village level. Villages were assigned to one of four groups: (a) a control arm, (b) HI, (c) LSM, and (d) HI + LSM. NMCP interventions and community engagement were used in all arms. A two-stage randomization process was used within each focal area, which took place in June 2015 at a community event in each focal area.

In the first stage of the randomization, a minimal sub-set of eligible villages was excluded from the treatment-arm allocation to reduce the risk of contamination from mosquito movement between different treatment arms [24]. Within each focal area, six potential randomization design options were identified, such that the remaining villages included in the trial for each option would form clusters of villages, and those clusters would be separated from each other by at least 800 m to reduce the potential for malaria mosquito movement between clusters [25]. At each community event in focal areas A and C, one of the six options was selected by drawing lots: six opaque folded cards corresponding to each of the six options were placed in a dish, and a volunteer from the community blindly selected one card. In focal area B, this stage was not necessary because the geography of the villages was such that excluding any of the villages would not have created any more clusters.

**Fig. 2:**
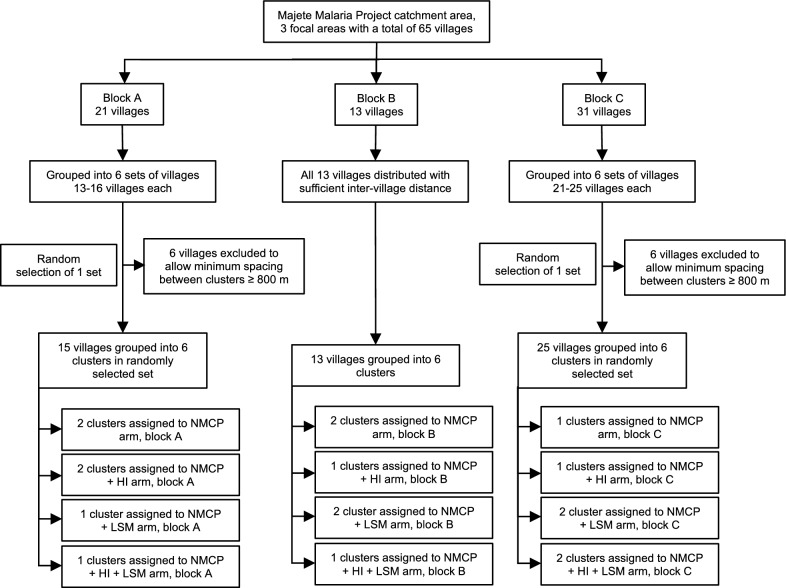
Trial profile showing two-stage randomization. Stage 1, randomization of villages in each focal area (block) into clusters. Stage 2, randomization of clusters into the four trial arms. *HI* house improvement, *LSM* larval source management, *NMCP *National Malaria Control Programme

In the second stage of the randomization, the unit of randomization was the cluster (Figs. 1, 2). At the community event in each focal area, the clusters were assigned to one of the four treatment arms by drawing lots, again using folded cards. All village residents and research staff were aware of the treatment assignments due to the nature of the intervention.

### Procedures

Two interventions were evaluated in this trial: HI and LSM. Both interventions were implemented using a community-driven approach, based on the expertise of The Hunger Project. Volunteers from all 65 villages in the Majete Malaria Project catchment area were trained as voluntary “health animators” by the project, covering a broad range of malaria topics [22] (Additional file 1). Most villages had one health animator who was selected by village leaders and guided by The Hunger Project using criteria of literacy skills, leadership potential and level of motivation [22]. In some cases, primarily based on village size, The Hunger Project and village leaders selected a second health animator for a village, so that there was a total of 77 health animators.

Larval source management in this trial consisted of draining, filling and larviciding. Water bodies were either drained or filled when feasible and if the community did not use the water for a designated purpose. All remaining water bodies were targets for larviciding with *Bacillus thuringiensis* subspecies *israelensis*, serotype H-14, strain AM65-52 (abbreviated *Bti*; commercial name VectoBac WDG, Valent Biosciences, Libertyville IL, USA). *Bti* has not been used in Malawi outside of research settings, but it is widely used for mosquito control globally. Health animators in villages assigned to LSM arms were given additional training on the concepts and practice of LSM. Communities implemented all LSM activities with leadership from health animators, village-level LSM committees, and community leaders (Additional file 1).

House improvement in this trial consisted of modifications to houses aimed at blocking entry by malaria vectors. Following discussions with communities, the agreed modifications consist of: closing all eaves (i.e., where a wall meets the overhang of the roof) using local material similar to that used to construct the house (i.e., bricks and extra mud for most houses); closing all holes in the wall not used for ventilation using the same materials used for closing eaves; covering windows and other openings used for ventilation with aluminium screens that allow airflow; and modifying doors so as to fully cover doorways when closed. Similar to LSM, health animators in villages assigned to HI arms were given additional training on the concepts and practices of HI, and communities implemented all HI activities with leadership from health animators, village-level HI committees and community leaders (Additional file 1).

Entomological indicators of malaria transmission were assessed by sampling for mosquitoes using Suna traps (Biogents AG, Regensburg, Germany), which use a synthetic blend of volatiles found on human skin and carbon dioxide to attract host-seeking *Anopheles* mosquitoes [26]. The standardized odour blend allows for reliable comparisons among trapping locations [27]. Suna traps were set at the houses of study participants for two consecutive nights (one indoors, one outdoors), and mosquitoes were collected from the traps each morning after a night of sampling. Information about the household was recorded through a standardized form, including the types of bed nets in the house, the use of any insecticides, and the presence of livestock. All collected mosquitoes were preserved using a desiccant and identified using standard morphological and molecular techniques (Additional file 1). Real-time PCR was used to assess the presence of *Plasmodium falciparum* parasites in the heads and thoraces of female *Anopheles* mosquitoes after removing the abdomens (Additional file 1).

Epidemiological data were collected using a household survey adapted from the internationally standardized malaria indicator survey, which included a core questionnaire covering demographic and socio-economic data, and an additional module covering malaria control intervention practices and morbidity indicators [28]. For children aged 6–59 months and women aged 15–49 years, the presence of *P. falciparum* parasites was measured by rapid diagnostic test (RDT; SD BIOLINE Malaria Ag P.f. HRP-II, Standard Diagnostics, Yongin-si, Republic of Korea), and haemoglobin levels were measured using HemoCue^®^ Hb 301 (HemoCue, Ängelholm, Sweden). These demographic groups were chosen to align with the groups sampled in the national malaria indicator surveys in Malawi prior to and during the study.

A repeated cross-sectional survey sampling framework was used for both epidemiological surveys and adult mosquito sampling, with slight differences in the household selection procedure between the baseline (April 2015 through April 2016) and trial periods (May 2016 through May 2018; Additional file 1). In both cases, a sample of households was selected from a demographic database covering the study area (Additional file 1) every 2 months for the epidemiological survey, and a subset of those households was randomly selected for adult mosquito sampling. Epidemiological surveys and adult mosquito sampling (for selected households) were conducted in these households over a 6- to 8-week period in each round.

### Outcomes

The primary outcome was the entomological inoculation rate (EIR) at the end of the trial period (January to May 2018). EIR was calculated as the product of the sporozoite rate and the number of host-seeking *Anopheles* mosquitoes collected per house over a defined period of time (Additional file 1). The EIR over the entire trial period (May 2016 through May 2018) and the difference between EIR in the trial period and EIR in the baseline period (April 2015 through April 2016) were also assessed as secondary outcomes. Other secondary outcomes were *Anopheles* mosquito density (indoors and outdoors; separately by species and pooled for all *Anopheles*), *P. falciparum* parasite prevalence (regardless of symptoms, and with symptoms) in children aged 6–59 months (with children aged 6–23 months also analysed as a subgroup) and women aged 15–49 years, and haemoglobin levels in the same age groups. These secondary outcomes were assessed in three ways: the end of the trial period; averaged over the entire trial period; and as the difference between intervention and baseline periods.

### Pre-trial power analysis

The catchment area was limited to the 65 villages described above at the onset of the Majete Malaria Project [29]. From these 65 villages, 53 villages grouped into 18 clusters were included in the trial following the randomized exclusion described above (Figs. 1, 2) [24]. For the pre-trial power analysis, annual EIR in the control arm was assumed to have a log-normal distribution with mean and standard deviation approximately 45 and 14, respectively, giving an effective range of approximately 0 to 100. It was further assumed that a clinically effective intervention would be one that halved the mean EIR, i.e., an effect size of 0.500. The resulting power to detect a clinically significant main effect of HI, testing at the conventional 5% level, was 0.669. The associated standard error of the estimated effect size was 0.286. This implied that a 95% confidence interval for the relative reduction in EIR associated with a clinically significant main effect would extend from 0.285 to 0.876. For the main effect of LSM, the corresponding figures were 0.728, 0.265 and a range from 0.298 to 0.840.

### Cluster-level analysis

The statistical model for the primary analysis was a randomized block ANOVA allowing for block effects plus main effects for each intervention and an interaction term, using a robust version of the ANOVA F-test that respected the restricted randomization. Differences were assessed at the cluster level, and every household was assumed to be fully covered by the interventions in the trial arm to which it was allocated (i.e., intention-to-treat). In addition to the primary outcome of EIR from January through May 2018, this primary analysis protocol was also used to assess all other outcomes (including EIR, mosquito density, parasite prevalence and haemoglobin levels) in the baseline period, the trial period, at the end of the intervention, and as the difference between the trial period minus baseline. The following distributional assumptions were used in the statistical models: log-normal for EIR; Poisson for mosquito density; binomial for parasite prevalence; and normal for haemoglobin.

### Household- and individual-level analysis

Differences were also assessed among the trial arms in mosquito density at the household level, and in parasite prevalence and haemoglobin levels at the individual level, to allow for estimation of household-level and individual-level covariate effects. A generalized linear mixed effects model was fitted to each outcome variable in relation to demographic, socio-economic, and environmental factors, and accounting for village and household-within-village random effects. Distributional assumptions for each outcome variable were the same as those used in the cluster-level analysis. All models included block effects (focal area A, B, or C) to account for unmeasured differences among the focal areas, as well as month of collection (from 1 = April 2015 to 38 = May 2018) to account for unmeasured differences over time.

When describing the mean EIR, 95% confidence intervals were calculated assuming a Poisson distribution to avoid zero-width confidence intervals produced by the log-normal assumption when the mean was zero.

### Statistical software

R version 3.5 was used for all analyses. R packages included mgcv, lme4 and INLA.

## Results

Out of 65 eligible villages in the trial catchment area, 12 villages were excluded from the treatment arm allocation to reduce the risk of contamination between different treatment arms (Figs. 1, 2). The remaining 53 villages were grouped into 18 clusters, which were assigned to the four trial arms in June 2015 (Fig. 2). The 53 villages comprised 4558 households and a population of 20,013 as of February 2015.

During five rounds in the baseline period (April 2015 through April 2016) and 12 rounds in the trial period (May 2016 through May 2018), 1380 and 3240 households, respectively, were selected for epidemiological surveys; in the trial period, some households were selected multiple times in different rounds. 1072 and 1844 unique household visits during baseline and trial periods, respectively, were included in the analysis presented here, including households that were replaced by the nearest neighbour when the selected household was absent. 42 visits (baseline period) and 118 visits (trial period) ended when the head of household did not consent, and a further 266 visits (baseline period) and 1098 visits (trial period) were to households without at least one child aged 6–59 months or woman aged 15–49 years. In the trial period, an additional 180 household visits were excluded from the presented analysis because they were made in villages excluded from treatment arm allocation. From the included visits during the baseline period, 894 RDT results were obtained from children aged 6–59 months, and 1161 RDT results were obtained from women aged 15–49 years. During the trial period, the corresponding figures were 1370 for children aged 6–59 months and 1935 for women aged 15–49 years (Additional file 1: Figure S2).

**Fig. 3:**
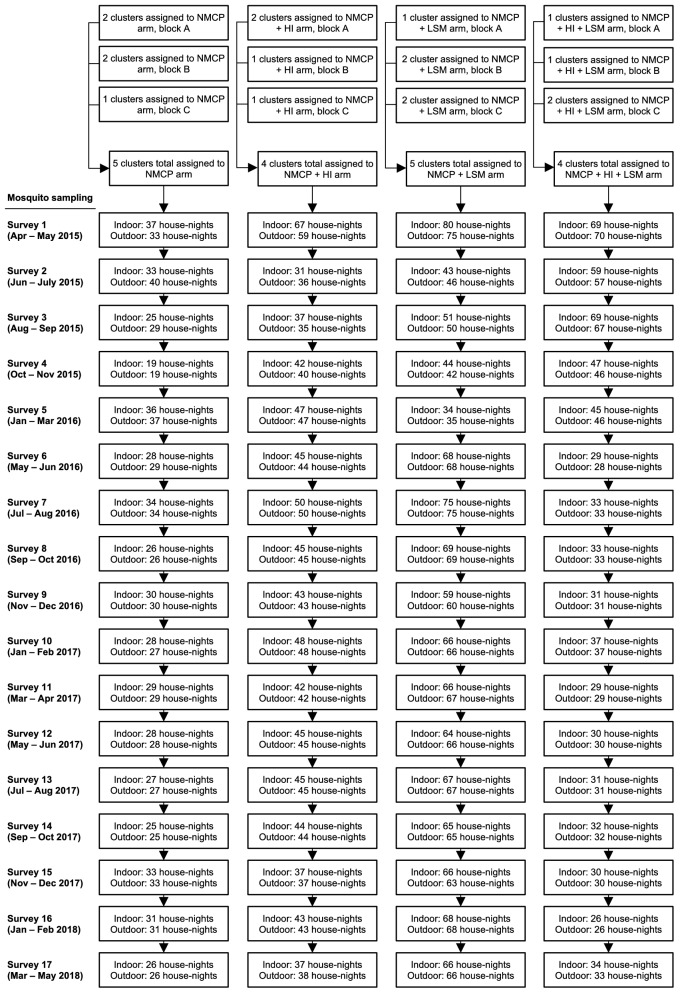
Trial profile showing entomological surveys. Number of house-nights conducting mosquito sampling indoors and outdoors in each round, by trial arm. *HI* house improvement, *LSM* larval source management, *NMCP *National Malaria Control Programme

Of the 1380 and 3240 households selected for epidemiological surveys, 1104 (80%) and 2340 (72%), respectively, were selected for adult mosquito sampling. In the analysis presented here, 1098 indoor and 1090 outdoor samples from the baseline period and 2043 indoor and 2042 outdoor samples from the trial period have been included (Fig. 3).

The trial design resulted in an unbalanced distribution of the study population among the four trial arms, with about 1.5 times as many households in the LSM arm as in each of the other three arms (Table 1). The baseline characteristics of the four trial arms are also shown in Table 1.

**Table 1:** Baseline characteristics of the four trial arms

	Control	HI	LSM	HI + LSM
Study cluster characteristics				
Clusters	5	4	5	4
Villages	7	13	24	9
Households	1056	1030	1520	952
Population	4244	4568	6801	4400
Household characteristics				
Median altitude of houses, m (range)	90 (66, 510)	200 (85, 632)	190 (73, 569)	225 (144, 550)
% households in the lowest socioeconomic category^a^ (95% CI)	16.3 (11.1, 21.6)	19.1 (14.6, 23.6)	14.5 (10.5, 18.4)	12.6 (9.0, 16.2)
% households with ≥ 1 long-lasting insecticidal nets (95% CI)	35.3 (28.5, 42.1)	28.8 (23.6, 34.1)	27.3 (22.3, 32.3)	30.5 (25.5, 35.5)
Children characteristics				
Median age, months (IQR; N)	31 (20, 44; 106)	30 (19, 48; 223)	33 (21, 47; 199)	30 (18, 44; 213)
Long-lasting insecticidal nets use (%) in children 6–59 months (95% CI)	43.4 (34.0, 52.8)	34.5 (28.3, 40.8)	29.1 (22.8, 35.5)	38.0 (31.5, 44.5)
Malaria infection prevalence (%) in children 6–59 months (95% CI; N)	26.4 (19.0, 35.5; 106)	26.5 (21.1, 32.6; 223)	38.7 (32.2, 45.6; 199)	44.6 (38.1, 51.3; 213)
Mean haemoglobin level in children 6–59 months, g/dL (95% CI; N)	10.9 (10.6, 11.1; 106)	10.7 (10.5, 10.9; 223)	10.2 (10.0, 10.5; 199)	10.2 (10.0, 10.4; 213)
Entomological characteristics				
Mean number of *Anopheles* females collected indoors per house per night (95% CI; N)	0.13 (0.07, 0.19; 150)	0.05 (0.02, 0.08; 224)	0.21 (0.15, 0.26; 252)	0.12 (0.08, 0.16; 289)
Mean number of *Anopheles* females collected outdoors per house per night (95% CI; N)	0.21 (0.14, 0.28; 158)	0.06 (0.03, 0.10; 217)	0.31 (0.24, 0.38; 248)	0.21 (0.15, 0.26; 286)
% *Anopheles* females with *P. falciparum* DNA in head/thorax (sporozoite rate)^b^ (95% CI; N)	5.7 (1.9, 15.4; 53)	0.0 (0.0, 12.9; 26)	7.0 (3.7, 12.8; 128)	9.6 (5.1, 17.2; 94)

*HI* house improvement, *LSM* larval source management

^a^Socioeconomic categories were based on ownership of household items, using principal component analysis to determine quintiles

^b^Sporozoite rate combined for all *Anopheles* species, combined for indoor and outdoor sampling

The proportion of households owning at least one ITN during the baseline period was 29% (95% CI 27%, 32%), and the proportion of children aged 6–59 months using an ITN was 34% (95% CI 31%, 37%). ITN ownership and use rose sharply to 89% (95% CI 84%, 92%) and 95% (95% CI 90%, 98%), respectively, following the NMCP’s mass distribution in April 2016, immediately prior to the start of the trial. Both ownership and use of ITNs fell gradually over the 2-year trial period, to 43% (95% CI 37%, 49%) and 54% (95% CI 44%, 63%), respectively, during the final round of data collection in March–April 2018. ITN use during the trial period was similar among the four trial arms (Table 2).

**Table 2:** Insecticide-treated bed net use among women and children during the trial period (May 2016–May 2018), by trial arm

Age group	Control	HI	LSM	HI + LSM
	Mean (%) (95% CI)	Mean (%) (95% CI)	Mean (%) (95% CI)	Mean (%) (95% CI)
Women 15–49 years	81.0 (76.3, 85.0)	79.0 (75.3, 82.3)	74.5 (71.3, 77.4)	82.0 (77.6, 85.7)
Children 6–59 months	85.6 (80.3, 89.7)	80.0 (75.9, 83.6)	76.9 (73.0, 80.4)	85.1 (80.1, 89.1)
Children 6–23 months	81.3 (71.3, 88.3)	80.5 (72.8, 86.4)	75.3 (68.4, 81.1)	83.8 (73.8, 90.5)

The mean number of female *Anopheles* mosquitoes per house per night decreased over the three years of data collection, from 0.12 indoors and 0.18 outdoors in the baseline period to 0.02 indoors and 0.05 outdoors in the last year of the trial period (Table 3). The vast majority of *Anopheles* mosquitoes collected over three years were either *Anopheles arabiensis* (67%) or *Anopheles funestus *sensu stricto (26%) (Additional file 1: Table S1). There were no differences among the treatment arms when assessing the entomological outcomes at the cluster level (Additional file 1: Table S2). At the household level, after accounting for other risk factors the number of *A. arabiensis* outdoors was lower in the HI + LSM arm compared to the control arm (rate ratio (RR): 0.14, 95% CI 0.02, 0.74; Table 4). However, the number of *A. arabiensis* indoors was higher in both the LSM arm (RR: 11.13, 95% CI 2.36, 60.3) and HI + LSM arm (RR: 7.77, 95% CI 1.68, 39.7) compared to the control arm. There were no other differences among the treatment arms when assessing the entomological outcomes at the household level.

**Table 3:** Outcomes pooled across the entire study area, over time

Outcomes	Baseline year mean value observed (95%CI)	Trial year 1 mean value observed (95%CI)	Trial year 2 mean value observed (95%CI)
EIR (indoors + outdoors)^a^	0.010 (0.006, 0.015)	0.011 (0.007, 0.016)	0.001 (0.000, 0.003)
All *Anopheles* females indoors^a^	0.119 (0.099, 0.140)	0.095 (0.077, 0.112)	0.023 (0.015, 0.032)
*A. arabiensis* females indoors^a^	0.101 (0.082, 0.120)	0.049 (0.036, 0.061)	0.005 (0.001, 0.009)
*A. funestus* females indoors^a^	0.010 (0.004, 0.016)	0.040 (0.029, 0.052)	0.013 (0.006, 0.020)
All *Anopheles* females outdoors^a^	0.178 (0.153, 0.203)	0.116 (0.096, 0.135)	0.047 (0.034, 0.060)
*A. arabiensis* females outdoors^a^	0.158 (0.134, 0.181)	0.068 (0.053, 0.083)	0.009 (0.003, 0.014)
*A. funestus* females outdoors^a^	0.006 (0.002, 0.011)	0.041 (0.030, 0.053)	0.035 (0.024, 0.046)
Prevalence positive malaria RDT (%), women 15–49 years	19.2 (17.0, 21.6)	8.6 (7.1, 10.4)	15.1 (13.0, 17.4)
Prevalence positive malaria RDT (%), children 6–59 months	33.8 (30.8, 36.9)	19.3 (16.8, 22.1)	22.4 (19.4, 25.8)
Prevalence positive malaria RDT (%), children 6–23 months	28.7 (23.8, 34.0)	13.3 (9.8, 17.8)	14.7 (10.7, 19.8)
Prevalence positive malaria RDT + fever/temp^b^ (%), women 15–49 years	5.2 (4.0, 6.6)	1.3 (0.8, 2.1)	2.5 (1.7, 3.6)
Prevalence positive malaria RDT + fever/temp^b^ (%), children 6–59 months	14.2 (12.1, 16.6)	5.8 (4.4, 7.5)	5.2 (3.7, 7.2)
Prevalence positive malaria RDT + fever/temp^b^ (%), children 6–23 months	12.3 (9.1, 16.5)	3.0 (1.5, 5.7)	1.3 (0.4, 3.7)
Hb, g/dL, women 15–49 years	11.87 (11.78, 11.97)	12.23 (12.15, 12.31)	12.62 (12.54, 12.71)
Hb, g/dL, children 6–59 months	10.52 (10.41, 10.62)	10.86 (10.76, 10.96)	11.14 (11.03, 11.25)
Hb, g/dL, children 6–23 months	10.17 (10.00, 10.35)	10.34 (10.17, 10.52)	10.81 (10.64, 10.98)

*RDT* rapid diagnostic test, *EIR* entomological inoculation rate, *Hb* haemoglobin level

^a^EIR and *Anopheles* mosquito densities are based on nightly rates

^b^Self-reported fever in the last 48 h or body temperature measured over 37.5 °C

**Table 4:** Entomological outcomes for each of the study arms, aggregated over the 2-year trial period

Outcomes^a^	Control	HI		LSM		HI + LSM	
	Mean value observed (95% CI)	Mean value observed (95% CI)	Estimated rate ratio^b^ (95% CI)	Mean value observed (95% CI)	Estimated rate ratio^b^ (95% CI)	Mean value observed (95% CI)	Estimated rate ratio^b^ (95% CI)
EIR (indoors + outdoors)	0.001 (0.000, 0.008)	0.000 (0.000, 0.004)	NA	0.009 (0.005, 0.015)	NA	0.011 (0.005, 0.021)	NA
All *Anopheles* females indoors	0.061 (0.035, 0.087)	0.015 (0.005, 0.026)	1.11 (0.16, 8.17)	0.084 (0.064, 0.104)	2.18 (0.51, 10.9)	0.093 (0.062, 0.124)	2.56 (0.45, 16.8)
*A. arabiensis* females indoors	0.020 (0.005, 0.035)	0.011 (0.002, 0.021)	2.83 (0.42, 21.8)	0.041 (0.027, 0.055)	11.13 (2.36, 60.3)	0.040 (0.020, 0.060)	7.77 (1.68, 39.7)
*A. funestus* females indoors	0.029 (0.011, 0.047)	0.004 (0.000, 0.009)	2.29 (0.21, 23.1)	0.035 (0.022, 0.048)	0.41 (0.04, 2.56)	0.048 (0.026, 0.070)	1.26 (0.23, 6.05)
All *Anopheles* females outdoors	0.058 (0.033, 0.083)	0.017 (0.006, 0.028)	0.51 (0.09, 2.80)	0.136 (0.111, 0.162)	1.95 (0.49, 8.41)	0.051 (0.028, 0.074)	0.36 (0.06, 2.08)
*A. arabiensis* females outdoors	0.043 (0.021, 0.065)	0.015 (0.005, 0.026)	0.95 (0.22, 3.97)	0.068 (0.049, 0.086)	0.91 (0.30, 2.94)	0.024 (0.008, 0.040)	0.14 (0.02, 0.74)
*A. funestus* females outdoors	0.009 (0.000, 0.019)	0.002 (0.000, 0.006)	0.38 (0.01, 8.67)	0.058 (0.041, 0.074)	3.49 (0.55, 28.8)	0.024 (0.008, 0.040)	2.12 (0.26, 22.2)

*EIR* entomological inoculation rate, *HI* house improvement, *LSM* larval source management, *NA* not applicable because EIR analysis at household level not done due to low values

^a^EIR and *Anopheles* mosquito densities are based on nightly rates

^b^Effect size estimates are presented as relative rate ratios based on household-level generalized linear models with the control arm as the reference category

**Fig. 4:**
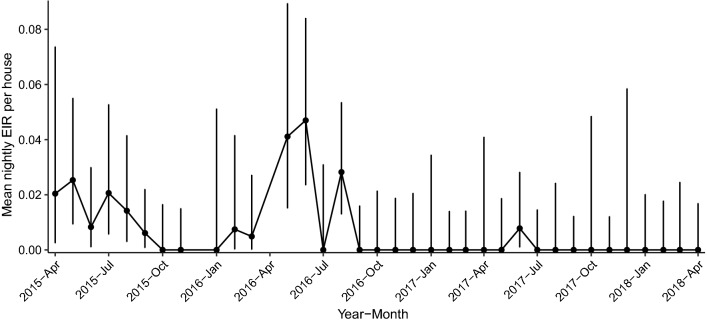
Entomological inoculation rate (EIR) during each month of sampling. EIR is shown as the nightly mean across the entire study site, with error bars showing the 95% confidence intervals

The mean nightly EIR in the baseline period was 0.010 infectious bites per person (95% CI 0.006–0.015) (Table 3), for an estimated annual EIR of 3.50. The EIR fluctuated annually, with seasonal peaks typically following the rainy season and, more broadly, declining over the 3-year period (Fig. 4). Strikingly, over the last 11 months of the trial period (8 June 2017 to 10 May 2018), the EIR was zero across all four trial arms, and therefore the primary outcome (EIR at the end of the trial period) could not be statistically assessed. There were no differences among the trial arms at the cluster level when comparing the EIR from the full trial period (p = 0.33; Additional file 1: Table S2). The mean nightly EIR was 0.001 infectious bites per person (95% CI 0.000–0.008) in the control arm, 0.000 (0.000–0.004) in the HI arm, 0.009 (0.005 to 0.015) in the LSM arm, and 0.011 (0.005–0.021) in the HI + LSM arm (Table 4).

The prevalence of *P. falciparum* infection (by positive malaria RDT) decreased from the baseline year to the first year of the trial in all three age categories measured, and remained below baseline levels in the second year of the trial (Table 3). A similar pattern was seen with the prevalence of symptomatic malaria (RDT positive with either fever in the last 48 h or temperature ≥ 37.5) in all three age groups. For example, in children aged 6–23 months, the prevalence of symptomatic malaria declined from 12.3% (95% CI 9.1%, 16.5%) at baseline to 1.3% (95% CI 0.4%, 3.7%) in the second year of the trial. There were no differences among the treatment arms when assessing the prevalence of malaria either with or without symptoms at either the cluster (Additional file 1: Table S2) or household level in all three age categories (Table 5). Haemoglobin levels increased over the three years of data collection in all three age categories (Table 3), but there were no differences in haemoglobin levels among the trial treatment arms at either the cluster (Additional file 1: Table S2) or household level (Table 6).

**Table 5:** Malaria prevalence outcomes for each of the study arms, aggregated over the 2-year trial period

	Control	HI		LSM		HI + LSM	
Outcomes	Mean value observed (95% CI)	Mean value observed (95% CI)	Estimated odds ratio^a^ (95% CI)	Mean value observed (95% CI)	Estimated odds ratio^a^ (95% CI)	Mean value observed (95% CI)	Estimated odds ratio^a^ (95% CI)
Prevalence positive malaria RDT (%), women 15–49 years	10.4 (7.5, 14.3)	7.8 (5.8, 10.4)	0.75 (0.37, 1.52)	14.9 (12.5, 17.6)	0.80 (0.41, 1.55)	10.2 (7.4, 13.8)	0.89 (0.41, 1.95)
Prevalence positive malaria RDT (%), children 6–59 months	18.1 (13.6, 23.8)	13.7 (10.7, 17.3)	1.38 (0.63, 3.03)	26.8 (23.1, 30.8)	1.80 (0.91, 3.60)	19.4 (14.9, 24.9)	1.95 (0.90, 4.31)
Prevalence positive malaria RDT (%), children 6–23 months	13.8 (7.9, 23.0)	5.5 (2.7, 10.9)	0.51 (0.09, 2.83)	20.1 (14.8, 26.7)	2.77 (0.79, 11.94)	10.8 (5.6, 19.9)	2.08 (0.43, 10.59)
Prevalence positive malaria RDT + fever/temp^b^ (%), women 15–49 years	2.2 (1.1, 4.5)	1.9 (1.1, 3.5)	0.75 (0.13, 4.10)	1.6 (0.9, 2.7)	0.59 (0.12, 2.97)	2.0 (1.0, 4.1)	0.73 (0.12, 4.57)
Prevalence positive malaria RDT + fever/temp^b^ (%), children 6–59 months	7.4 (4.6, 11.7)	5.0 (3.3, 7.6)	0.63 (0.21, 1.84)	4.6 (3.1, 6.8)	0.76 (0.29, 2.10)	6.2 (3.8, 10.0)	0.70 (0.24, 2.05)
Prevalence positive malaria RDT + fever/temp^b^ (%), children 6–23 months	5.0 (2.0, 12.2)	1.6 (0.4, 5.5)	0.02 (0.00, 1.20)	2.3 (0.9, 5.8)	0.19 (0.00, 4.53)	1.4 (0.2, 7.3)	0.05 (0.00, 2.61)

*HI* house improvement, *LSM* larval source management, *RDT* rapid diagnostic test

^a^Effect size estimates are presented as odds ratios based on individual-level generalized linear models with the control arm as the reference category

^b^Self-reported fever in the last 48 h or body temperature measured over 37.5 °C

**Table 6:** Haemoglobin levels for each of the study arms, aggregated over the 2-year trial period

	Control	HI		LSM		HI + LSM	
Outcomes	Mean value observed (95% CI)	Mean value observed (95% CI)	Estimated effect size^a^ (95% CI)	Mean value observed (95% CI)	Estimated effect size^a^ (95% CI)	Mean value observed (95% CI)	Estimated effect size^a^ (95% CI)
Hb, g/dL, women 15–49 years	12.31 (12.15, 12.47)	12.61 (12.49, 12.72)	− 0.10 (− 0.42, 0.21)	12.21 (12.10, 12.31)	− 0.11 (− 0.37, 0.15)	12.46 (12.31, 12.61)	0.01 (− 0.30, 0.33)
Hb, g/dL, children 6–59 months	10.80 (10.59, 11.01)	11.29 (11.16, 11.42)	0.17 (− 0.22, 0.56)	10.68 (10.55, 10.81)	− 0.02 (− 0.34, 0.31)	11.08 (10.89, 11.27)	0.28 (− 0.15, 0.71)
Hb, g/dL, children 6–23 months	10.61 (10.26, 10.96)	10.86 (10.65, 11.07)	0.08 (− 0.44, 0.61)	10.14 (9.93, 10.35)	− 0.40 (− 0.90, 0.10)	10.61 (10.28, 10.95)	− 0.14 (− 0.70, 0.42)

*Hb* haemoglobin level, *HI* house improvement, *LSM* larval source management

^a^Effect size estimates are based on individual-level generalized linear models with the control arm as the reference category

## Discussion

This trial assessed the potential for community-based HI and/or LSM to reduce malaria parasite transmission and prevalence beyond the level of control afforded by high coverage of NMCP interventions combined with community engagement. The choice of EIR as the primary outcome was based on the assumption that a measurable EIR would be more sensitive than epidemiological outcomes to changes effected by the vector control interventions being investigated [30]. Critically, the EIR decreased from about 3.5 infectious bites per person per year at baseline to zero across all four trial arms by the last year of the trial. This signalled a promising and important reduction in malaria transmission for the area, although EIR values of zero prevented the pre-planned assessment of differences in EIR among the trial arms. The secondary outcomes included vector mosquito densities, malaria prevalence with and without symptoms, and haemoglobin levels. Most of these outcomes provided no evidence that either community-based HI, LSM or the combination of HI and LSM had an effect at either the village or household level. The exceptions to this were in the household-level analysis: there were fewer *A. arabiensis* outdoors in the HI + LSM arm compared to the control; but there were also more *A. arabiensis* indoors in the LSM and HI + LSM arms compared to the control. The observed decline in EIR to below the level of detection over the 2-year trial period was likely due to the high rates of ITN use following a mass ITN distribution and intensive community engagement across the entire study site to promote the NMCP control strategy. This reduction in EIR was accompanied by reductions in the prevalence of positive RDTs and the prevalence of symptomatic malaria in all three measured age groups, as well as an increase in haemoglobin levels. Overall, in this context, there was no statistical evidence that community-based HI and/or LSM contributed to further reductions in malaria parasite transmission or prevalence beyond the reductions provided by the mass ITN distribution, other NMCP interventions, and the community engagement programme.

One limitation of the study was that the baseline EIR for the study site was 10 times lower than expected based on previous data from the region [21], and decreased to zero across the study site by the end of trial, limiting the statistical usefulness of this outcome for measuring differences in malaria transmission among the trial arms. Owing to the time required for organizing the logistical infrastructure associated with community implementation of the trial interventions, the pre-trial power analysis was conducted before baseline data collection and was, therefore, based on previous data rather than data from the baseline year. Ultimately, the trial had lower power to assess the primary outcome than was designed, but the trial design could not be altered retroactively. However, the measurement of EIR during this study captured an important reduction in malaria parasite transmission over time, and changes over the study period in asymptomatic and symptomatic malaria parasite prevalence as well as haemoglobin levels in women and children corroborated the observed EIR values. Additionally, the unique sampling strategy (Additional file 1) used in this study allowed fine-scale spatio-temporal variation to be captured in both entomological and epidemiological outcomes over three years of data collection and these spatio-temporal patterns to be compared in greater detail (Amoah et al. under review).

In settings with a very high annual EIR (above 15 infectious bites/person/year), EIR is more sensitive than malaria parasite prevalence to changes in transmission [31, 32], and, therefore, the assumed annual EIR of 45 infectious bites/person/year prior to starting this study partially guided the choice of EIR as the primary outcome [29]. The lower than expected baseline EIR values were likely due to increases in ITN ownership and use from 2000 to 2012, with the most recent mass ITN distribution prior to the study taking place in 2012 [33]. Similar reductions in malaria vector densities and EIR have occurred across many areas of Africa since the widespread scale up of ITNs for malaria control [34–36], and contemporaneous studies in the current study site found similarly low vector densities using a wide range of mosquito sampling methods [27, 37–39]. Measuring malaria intervention effects through entomological monitoring remains important for understanding the contributions of species-specific ecologies to variations in intervention impact. But more efficient methods for mosquito sampling or novel indicators of exposure to malaria vector bites (e.g., serological markers) are needed for measuring the impact of complementary interventions as study sites move toward lower transmission intensities.

This was the first trial of LSM or HI as community-based interventions. Previous studies on HI (and in some cases LSM) with highly controlled implementation have shown the efficacy of these interventions against malaria indicators [15, 17]. In the current study, a new component was added to the interventions by structuring the implementation through a community-based approach, which could provide a feasible and sustainable path to scale-up of the interventions. With this emphasis on community implementation, intervention coverage was dependent on community buy-in to the Majete Malaria Project. Consequently, in HI- and LSM-assigned villages where community buy-in to the project was low, coverage with the assigned interventions may have been lower than required for community-level effectiveness.

Previous controlled studies of LSM for malaria control have shown mixed results because of variation in the epidemiological context, larval ecology, and implementation strategies [17]. For example, where LSM has reduced malaria case incidence or parasite prevalence, ITN coverage has been less than 50% (or not reported). As far as the authors are aware, this is the first study to test the effect of LSM in the context of high ITN coverage following a national mass distribution that reached nearly 90% of households. In principle, LSM would have had an additional impact on malaria transmission by killing the outdoor biting mosquitoes that are not affected by ITNs. However, the high coverage with ITNs during most of the trial period apparently reduced malaria vector populations to nearly unmeasurable levels in all study arms, reducing the power to detect any incremental effect of LSM. A second potential explanation for these findings is that the zone of LSM implementation, which extended to 400 m past each LSM village border, was not large enough, and a sufficient number of malaria vectors from larval habitats outside the implementation zone entered the LSM villages to sustain malaria transmission levels. The 400 m distance was based on published records of mosquito dispersal distance and accounting for the relatively high human population density of the trial clusters [25], but mosquito dispersal can also vary considerably based on local weather conditions, land use, and natural or artificial barriers. Coverage of LSM is also a critical component of its success. Most previous studies of LSM have been conducted in areas where the habitats are few, fixed, and findable, a description that the World Health Organization continues to use for the current guidelines on LSM [17]. In the current study, the LSM committees were able to map and track the larval habitats in and around their villages (i.e., habitats were few and findable). Furthermore, quarterly checks on larvicide effectiveness showed the expected impact of *Bti* spraying on mosquito larvae, indicating that community-based spray teams are capable of sufficient *Bti* application quality on habitats that they sprayed. However, spray team weekly visits to every habitat were not verified, and there remains a possibility that poor coverage of LSM, due to missed habitats, explains the results. An analysis of LSM committee and community LSM practices will be reported separately, providing better evidence for whether a decentralized approach to managing LSM leads to sufficient coverage for the intervention.

Two previous randomized controlled trials have suggested that HI can reduce malaria transmission [15, 16]. A recent systematic review and meta-analysis of modern versus traditional houses, including 84 observational and six intervention studies, found that housing is likely an important risk factor for malaria [40], a finding that was further supported by a recent multi-country analysis of survey data [14]. The current study did not find evidence for HI reducing malaria transmission or prevalence. As with LSM, high coverage with ITNs across the study site may have reduced the power to detect any supplemental effect of HI. Another likely explanation for these findings may be imperfect HI coverage in villages where community buy-in to the project was low, or where houses completing HI had gaps remaining in the eaves, windows or doors through which mosquitoes may have entered. For HI to be effective for reducing malaria transmission, all openings for mosquito entry on a house should be completely screened or completely closed, which effectively reduces indoor malaria vector densities [37]. Future work should assess HI implementation strategies that better manage HI quality while still giving the communities ownership over the process. Also, future studies are needed to understand the effect of different house designs on mosquito entry and malaria transmission as well as the acceptability, feasibility, and sustainability of those designs in communities where they would be implemented [41].

## Conclusions

This trial was conducted in the context of a mass ITN distribution and an intensive community engagement project, with high ITN use leading to markedly reduced malaria parasite transmission and prevalence in the entire study area, including in the control and intervention arms. Notably, the EIR across the four trial arms decreased to zero over 2 years. In this setting, community-based LSM and/or HI did not provide any further reduction in malaria transmission or prevalence beyond the level reached in the control arm. Other studies, with higher malaria transmission intensities and lower ITN use than in this study area in southern Malawi, have shown that LSM and HI are efficacious malaria interventions [17, 40]. In settings with very low transmission resulting from high ITN use and effective treatment, novel evaluation strategies are required to demonstrate the impact of LSM and HI as complementary interventions, with the potential to cause further reductions in malaria transmission and move towards elimination of malaria.

## Supplementary Information

**Additional file 1. **Expanded methods and results.

## Data Availability

All of the individual, de-identified participant data collected during the trial (including data dictionaries) will be shared. Additional documents that will be available are the study protocol, statistical analysis plan and analytic code. The data will be available beginning 2 years following article publication; to researchers who provide a methodologically sound proposal; for any purpose. Proposals should be directed to m.vanvugt@amsterdamumc.nl; to gain access, data requestors will need to sign a data access agreement.

## Abbreviations

95% CI: 95% Confidence interval
*Bti*: *Bacillus thuringiensis* subspecies *israelensis*
EIR: Entomological inoculation rate
Hb: haemoglobin level
HI: House improvement
IRS: Indoor residual spraying
ITN: Insecticide-treated bed net
LSM: Larval source management
NMCP: National Malaria Control Programme
RDT: Rapid diagnostic test
rMIS: Rolling malaria indicator survey
RR: Rate ratio
