# Supplementary material for: The effect of community-driven larval source management and house improvement on malaria transmission when added to the standard malaria control strategies in Malawi: a cluster-randomized controlled trial
Source: Malar J. 2021 May 22;20:232. doi: 10.1186/s12936-021-03769-0 (PMC8140568; doi:10.1186/s12936-021-03769-0)
Supplement: Supplementary file 1 — Additional file 1. Expanded methods and results. [file 12936_2021_3769_MOESM1_ESM.pdf]

Supporting information

Methods

Procedures

Health animator approach

We evaluated two interventions in this trial: LSM and HI. Both interventions were implemented using a community-driven approach, based on the expertise of THP. An essential component of this approach was empowering the community through a process of mindset change, leadership, vision, commitment and action. In brief, this meant that the community should perceive malaria as a challenge to be actively addressed, providing a basis for community action planning towards malaria control. Volunteers from all 65 villages in the MMP catchment area (slightly more than one per village on average) were trained as "health animators" by MMP. The training covered a broad range of malaria topics, and starting in November 2014 (17 months before the beginning of the trial), health animators led fortnightly malaria workshops in their communities to encourage the use of existing NMCP malaria interventions, and to set the foundation for the community-based implementation of the trial interventions.[1] Fortnightly malaria workshops continued in all 65 villages through May 2018 (the end of the trial; Fig. S1).

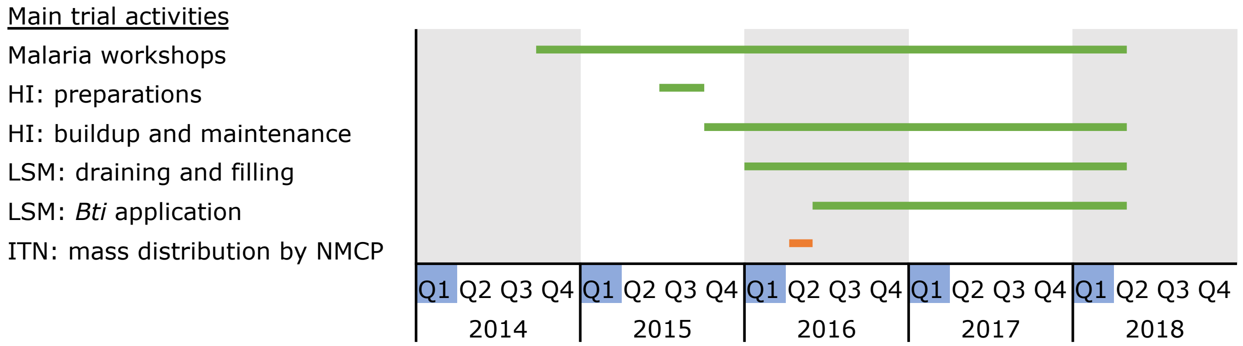

Fig. S1. Study timeline showing main trial activities by quarter from 2014 to 2018. Blue shading indicates the rainy season. Bti = Bacillus thuringiensis israelensis. HI = house improvement. ITN = insecticide-treated bed nets. LSM = larval source management. NMCP = National Malaria Control Programme. Q = quarter.

Larval source management implementation

Larval source management in this trial consisted of habitat modification and larviciding. Habitat modification referred to either draining or filling water bodies with the aim of permanently eliminating *Anopheles* larval habitats in cases where this was feasible and the water was not used by the community for a designated purpose. All remaining water bodies were targets for larviciding, i.e. spraying the water bodies with *Bacillus thuringiensis* subspecies *israelensis*, serotype H-14, strain AM65-52 (abbreviated *Bti*; commercial name VectoBac WDG, Valent Biosciences, Libertyville IL, USA). The bacterium *Bti* produces insecticidal crystal proteins that are toxic to mosquitoes and other closely related taxa.[2]

Health animators in villages assigned to LSM arms were given additional training on the concepts and practice of LSM. From January 2016 through May 2018, health animators in LSM villages led monthly workshops in their communities to discuss the concepts and practices of LSM. Each LSM village formed an LSM committee of ten to twelve members tasked with organising all LSM activities in the village. Health animators, LSM committees and community leaders encouraged all community members to participate in draining and filling potential larval habitats, with no material inputs provided by the project. Draining and filling started in January 2016 and continued through May 2018 (Fig S1). LSM committee members and health animators received additional training to implement *Bti* application in their respective villages. Equipment and material for *Bti* application were provided by the project. Weekly application of 300 g/ha *Bti* by LSM committees started in June 2016 and continued through May

2018 (Fig S1) for all water bodies that remained after draining and filling in LSM villages, including water bodies within 400 m of the village border as marked by the last house.

House improvement implementation
House improvement in this trial consisted of modifications to houses aimed at blocking entry by malaria vectors. Following discussions with communities, the agreed modifications consist of: closing all eaves (i.e. where a wall meets the overhang of the roof) using local material similar to that used to construct the house (i.e. bricks and extra mud for most houses); closing all holes in the wall not used for ventilation using the same materials used for closing eaves; covering windows and other openings used for ventilation with aluminium screens that allow airflow; and modifying doors so as to fully cover doorways when closed.

Similar to LSM, health animators in villages assigned to HI arms were given additional training on the concepts and practices of HI. From July to December 2015, health animators in HI villages led nine workshops in their communities to discuss the concepts and practices of HI. Each HI village formed an HI committee of ten to twelve members tasked with organising all HI activities in the village. Health animators, HI committees and community leaders encouraged heads of households in their villages to carry out any necessary improvements on their own houses. When household members were unable to improve their houses on their own, HI committees assisted them. Materials provided by the project for HI were aluminium screening (allocated to each household based on surface area to cover, and distributed and managed by HI committees) and a set of basic hand tools shared and managed by the HI committees. With organizational guidance from health animators and HI committees, communities prepared bricks for filling large eave openings from July to September 2015. Communities initially implemented HI from October through December 2015, with ongoing maintenance through May 2018 (Fig S1). Corrective actions were implemented in September 2016 following field assessment that showed poor-quality closure of eaves, and again in April 2017 after problems with corrosion of the aluminium screening.[1]

Sampling framework for surveys
All residents of the study area were enumerated between August 2014 and February 2015 to establish a demographic surveillance system, using a combination of Open Data Kit (ODK) and OpenHDS software.[3,4] Information was updated in OpenHDS during each round of epidemiological surveys for households in the selected sample for that round, plus any households that were replaced by the nearest neighbour when the selected household was absent. We used a repeated cross-sectional survey sampling framework for both epidemiological surveys and adult mosquito sampling, with slight differences in the household selection procedure between the baseline (April 2015 through April 2016) and intervention periods (May 2016 through May 2018). In the first round of the baseline period, 300 households were selected for the epidemiological survey using randomized inhibitory spatial sampling, which allows for efficient spatial predictions and estimation of covariance structure while still including a probability sampling frame.[5] In the second round, 270 households were selected using the same procedure. For each of the three subsequent rounds of the baseline period, 270 households were selected using adaptive geostatistical design.[6] For the five baseline rounds, 75% of the households on the epidemiological survey list were randomly selected for adult mosquito sampling. In the baseline period, previously sampled households were not eligible for sampling in subsequent rounds. In the trial period, 270 households were selected every two months for the epidemiological survey using randomized inhibitory spatial sampling, and 195 of those households were randomly selected for adult mosquito sampling. Households from villages excluded from the trial treatment allocation were included in the surveys. All households were eligible for selection in each round of the trial period regardless of whether they were selected in a previous round. Epidemiological surveys and adult mosquito sampling (for selected households) were conducted at the 270 households over a six- to eight-week period.

PCR for species ID and P. falciparum presence
All collected mosquitoes were first identified using standard morphological techniques.[7,8] Those mosquitoes identified as *An. gambiae* s.l. or *An. funestus* s.l. were further identified according to standard molecular

techniques.[9,10] To assess the presence of *P. falciparum* parasites in the heads and thoraces of female *Anopheles* mosquitoes, we first removed the abdomen from the head and thorax. The head and thorax were then subjected to qPCR.[11,12] Specimens with a Ct value below 37.0 were considered positive for *P. falciparum*.

Outcomes

Calculation of EIR

The primary outcome was the entomological inoculation rate (EIR) at the end of the intervention period (January to May 2018). EIR is an indicator of malaria transmission and was calculated as the product of the sporozoite rate and the number of host-seeking *Anopheles* mosquitoes collected per house over a defined period of time. For calculation of EIR, indoor and outdoor mosquito samples were pooled. EIR was calculated for each mosquito species, and then summed across species, to get the total EIR within each cluster. For each species, the mean number of mosquitoes collected per Suna trap per night was multiplied by the sporozoite rate for that species. The sporozoite rate was the proportion of mosquitoes (in the given species and cluster) for which the head/thorax was positive for *P. falciparum* DNA as assessed by qPCR.

Results

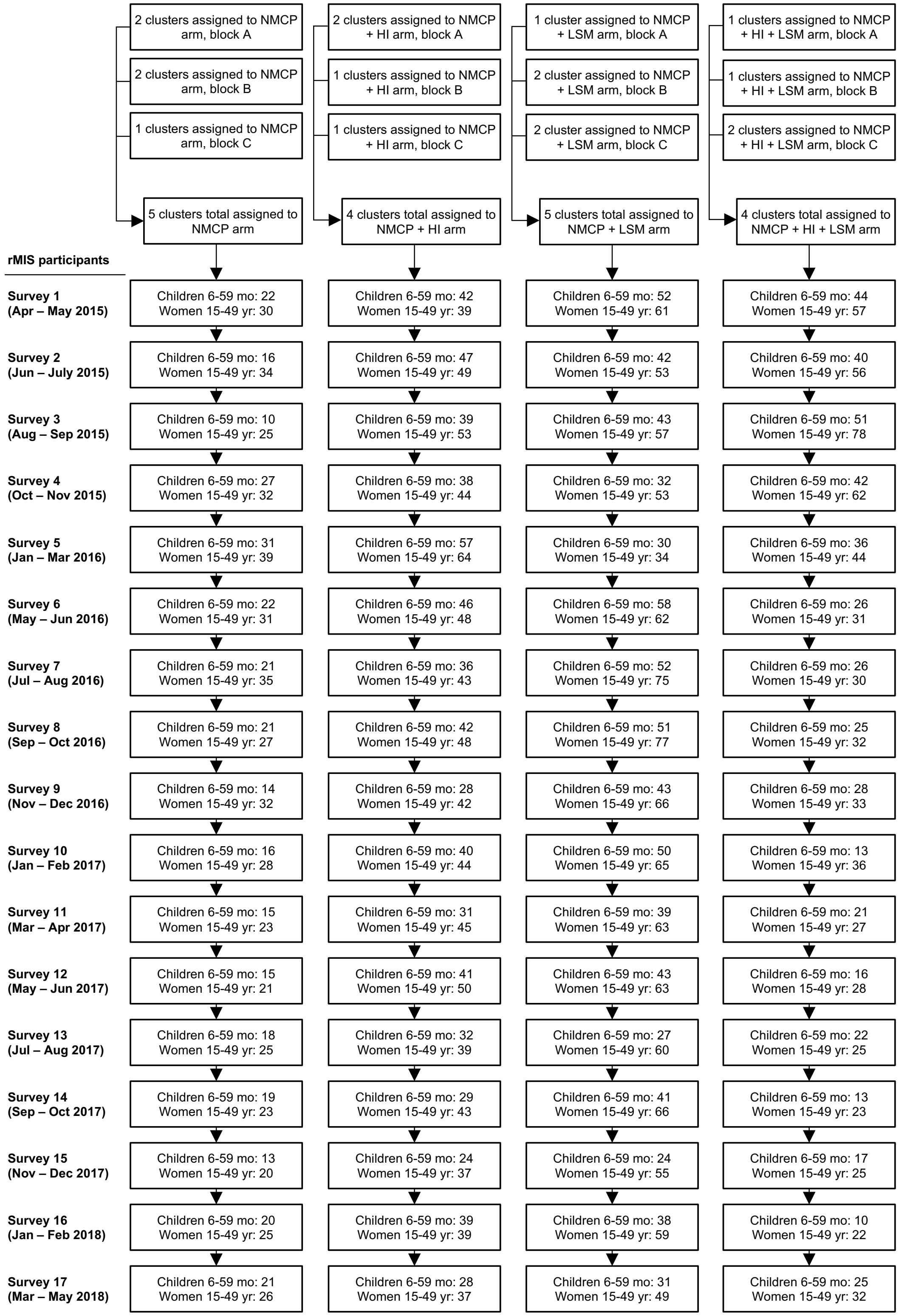

Fig. S2 Trial profile showing epidemiological surveys. Number of study participants in each round of the rolling malaria indicator survey (rMIS), by trial arm. HI = house improvement. LSM = larval source management. NMCP = National Malaria Control Programme.

Table S1. Total number of female *Anopheles* mosquitoes collected indoors and outdoors over the three-year study, shown by species, along with the sporozoite rate for each species.

Species	Number Collected Indoors	Number Collected Outdoors	sporozoite rate†
An. arabiensis	175	263	5.48%
An. funestus s.s.	74	96	11.17%
An. gambiae s.s.	5	6	18.18%
An. quadriannulatus	1	3	0.00%
*An. gambiae s.l.**	12	13	12.00%
*An. funestus s.l.***	4	5	11.11%
Total	271	386	7.46%

**An. gambiae s.l.* and ***An. funestus s.l.* were morphologically identified as belonging to the *An. gambiae* species complex and *An. funestus* species group, respectively, but could not be further identified by PCR.
†Percent of each species with *P. falciparum* DNA in head/thorax, combined for indoor and outdoor sampling

Table S2. Cluster-level analysis of primary and secondary outcomes showing p-values for data during the baseline period (April 2015 through April 2016), full trial period (May 2016 through May 2018), end of the trial period (January to May 2018), and the difference between the intervention period and the baseline period.

Outcome	Baseline	Trial	End trial	Difference Trial-Baseline
Nightly EIR (indoors + outdoors)	0.21	0.33	-	0.93
Total *Anopheles* indoors	0.55	0.34	0.81	0.73
An. arabiensis indoors	0.42	0.26	0.41	0.53
An. funestus indoors	0.29	0.37	0.75	0.84
Total *Anopheles* outdoors	0.11	0.14	0.23	0.47
An. arabiensis outdoors	0.08	0.26	0.85	0.22
An. funestus outdoors	0.61	0.03	0.30	0.09
Prevalence positive malaria RDT (%), women 15-49 y	0.92	0.87	0.15	0.92
Prevalence positive malaria RDT (%), children 6-59 m	0.71	0.72	0.47	0.90
Prevalence positive malaria RDT (%), children 6-23 m	0.70	0.39	0.50	0.86
Prevalence positive malaria RDT + fever/temp* (%), women 15-49 y	0.93	0.87	0.15	0.92
Prevalence positive malaria RDT + fever/temp* (%), children 6-59 m	0.72	0.73	0.47	0.89
Prevalence positive malaria RDT + fever/temp* (%), children 6-23 m	0.83	0.40	0.48	0.85
Hb, g/dL, women 15-49 y	0.40	0.71	0.56	0.61
Hb, g/dL, children 6-59 m	0.14	0.52	0.70	0.03
Hb, g/dL, children 6-23 m	0.14	0.18	0.93	0.31

*self-reported fever in the last 48 hrs or body temperature measured over 37.5 ℃

References

1 van den Berg H, Van Vugt M, Kabaghe AN, *et al.* Community-based malaria control in southern Malawi: a description of experimental interventions of community workshops, house improvement and larval source management. *Malar J* 2018; **17**: 1–12.

2 Charles J-F, Nielsen-LeRoux C. Mosquitocidal bacterial toxins: diversity, mode of action and resistance phenomena. *Mem Inst Oswaldo Cruz* 2000; **95**: 201–6.

3 Homan T, Di Pasquale A, Kiche I, *et al.* Innovative tools and OpenHDS for health and demographic surveillance on Rusinga Island, Kenya. *BMC Res Notes* 2015; **8**: 397.

4 McCann RS, van den Berg H, Diggle PJ, *et al.* Assessment of the effect of larval source management and house improvement on malaria transmission when added to standard malaria control strategies in southern Malawi: study protocol for a cluster-randomised controlled trial. *BMC Infect Dis* 2017; **17**: 639.

5 Chipeta M, Terlouw D, Phiri K, Diggle P. Inhibitory geostatistical designs for spatial prediction taking account of uncertain covariance structure. *Environmetrics* 2016; : 1–11.

6 Chipeta MG, Terlouw DJ, Phiri KS, Diggle PJ. Adaptive geostatistical design and analysis for prevalence surveys. *Spatial Statistics* 2016; **15**: 70–84.

7 Gillies MT, DeMeillon B. The Anophelinae of Africa South of the Sahara (Ethiopian zoogeographical region). In: Johannesburg: South Afric Instit for Med Res. 1968.

8 Gillies MT, Coetzee M. A supplement to the Anophelinae of Africa south of the Sahara (Afrotropical Region). Johannesburg, South Africa: South African Institute for Medical Research, 1987.

9 Scott JA, Brogdon WG, Collins FH. Identification of single specimens of the *Anopheles gambiae* complex by the polymerase chain reaction. *Am J Trop Med Hyg* 1993; **49**: 520–9.

10 Koekemoer LL, Kamau L, Hunt RH, Coetzee M. A cocktail polymerase chain reaction assay to identify members of the *Anopheles funestus* (Diptera: Culicidae) group. *Am J Trop Med Hyg* 2002; **66**: 804–11.

11 Bass C, Nikou D, Blagborough AM, *et al.* PCR-based detection of Plasmodium in Anopheles mosquitoes: a comparison of a new high-throughput assay with existing methods. *Malar J* 2008; **7**: 177–9.

12 Perandin F, Manca N, Calderaro A, *et al.* Development of a Real-Time PCR Assay for Detection of Plasmodium falciparum, Plasmodium vivax, and Plasmodium ovale for Routine Clinical Diagnosis. *J Clin Microbiol* 2004; **42**: 1214–9.
